# Morgagni Hernia in Down Syndrome: A Case Report

**DOI:** 10.7759/cureus.48019

**Published:** 2023-10-31

**Authors:** Aumniyat S Alrashidi, Muhanned A Amawi, Nouf O Alanazi, Dina A Aljohani, Ghadah A Alanazi, Manal M Alatawi

**Affiliations:** 1 Pediatrics Department, Faculty of Medicine, University of Tabuk, Tabuk, SAU; 2 Pediatrics Department, King Salman Armed Forces Hospital, Tabuk, SAU

**Keywords:** morgagni hernia, down syndrome, congenital, respiratory distress, computed tomography (ct )

## Abstract

Morgagni hernia is a rare form of congenital diaphragmatic hernia. It is associated with other congenital abnormalities. Its association with Down syndrome has been reported in the literature. While pediatric patients usually present with respiratory manifestations, the clinical presentation of Morgagni hernia is non-specific. Therefore, Morgagni hernia is mainly diagnosed by radiological imaging. The rarity of this type of hernia along with the vague clinical presentation can lead to missed diagnosis. Here, we report the case of a Down syndrome patient with an acute onset of shortness of breath. She was diagnosed with Morgagni hernia by computed tomography. Morgagni hernia should be considered in the differential diagnosis of Down syndrome patients presenting with respiratory distress.

## Introduction

Morgagni hernia (MH) is a rare diaphragmatic hernia. The incidence of MH in infants and children is low, accounting for about 1-5% of all types of congenital diaphragmatic hernias [1-3]. This hernia can allow the entry of the omentum and colon into the thoracic cavity. The stomach, liver, and small intestines can herniate as well [4,5]. There are no specific physical findings to suggest the diagnosis of MH. Patients may present with recurring respiratory distress or failure to thrive in infancy. However, in some cases, MH is diagnosed incidentally by imaging later in adulthood. The rarity of this hernia and its variable presentations make its diagnosis challenging. Hence, the diagnosis of MH is routinely confirmed radiologically [6]. Surgery is the main line of treatment for MH. Various surgical approaches have been described in the literature, including thoracic repairs by way of median sternotomy, thoracotomy, and thoracoscopy, in addition to abdominal approaches via laparotomy and laparoscopy [5].

A relationship has been considered between MH and developmental disorders including pentalogy of Cantrell, Down syndrome (DS), Turner syndrome, and Noonan syndrome [5,7]. Here, we report the case of a 10-year-old DS female with a history of bronchial asthma. She had an acute onset of shortness of breath. A routine chest X-ray showed a suspicious mass extending from the abdomen to the chest. However, computed tomography (CT) confirmed the suspected mass was MH.

## Case presentation

A 10-year-old DS Saudi girl from Tabuk, with a history of bronchial asthma, small patent ductus arteriosus, hypothyroidism, and morbid, obesity was brought to the emergency department in the early morning by her family with complaints of shortness of breath. According to her parents, she was doing well until the morning when she suddenly experienced shortness of breath that was aggravated by crying. The shortness of breath was accompanied by a runny nose, cough, and non-projectile vomiting. The vomiting contained only stomach content.

The patient had frequent lower abdominal pain that was described by her mother as being continuous, severe, sometimes associated with constipation, and relieved by laxatives. She had no other manifestations, and her past and family histories were unremarkable.

The patient was delivered via an emergency cesarean section, was diagnosed with DS by karyotyping, and was admitted to the neonatal intensive care unit until she was stable. During the first seven months of her life, she was on oxygen even at home.

A complete physical examination showed that the patient had typical DS characteristics. She was fully conscious, distressed, and was connected to an oxygen facemask. Her vital data were a pulse rate of 88 beats/minute, blood pressure of 107/55 mmHg, respiratory rate of 28 cycles/minute, and oxygen saturation of 93-99% in room air. Head and neck examination revealed macroglossia, enlarged tonsils, and lymphadenopathy. Chest auscultation showed decreased air entry mainly on the right side and diffuse wheezing mainly on the left side. Cardiac and abdominal examinations were unremarkable. The patient’s weight and height were 49 kg and 120 cm (5th-95th percentiles are 24.5-48 kg and 127-150 cm, respectively).

The patient had an asthma exacerbation and was given the usual management protocol. Endocrinology, ear, nose, and throat (ENT) and cardiology physicians were consulted.

**Figure 1:**
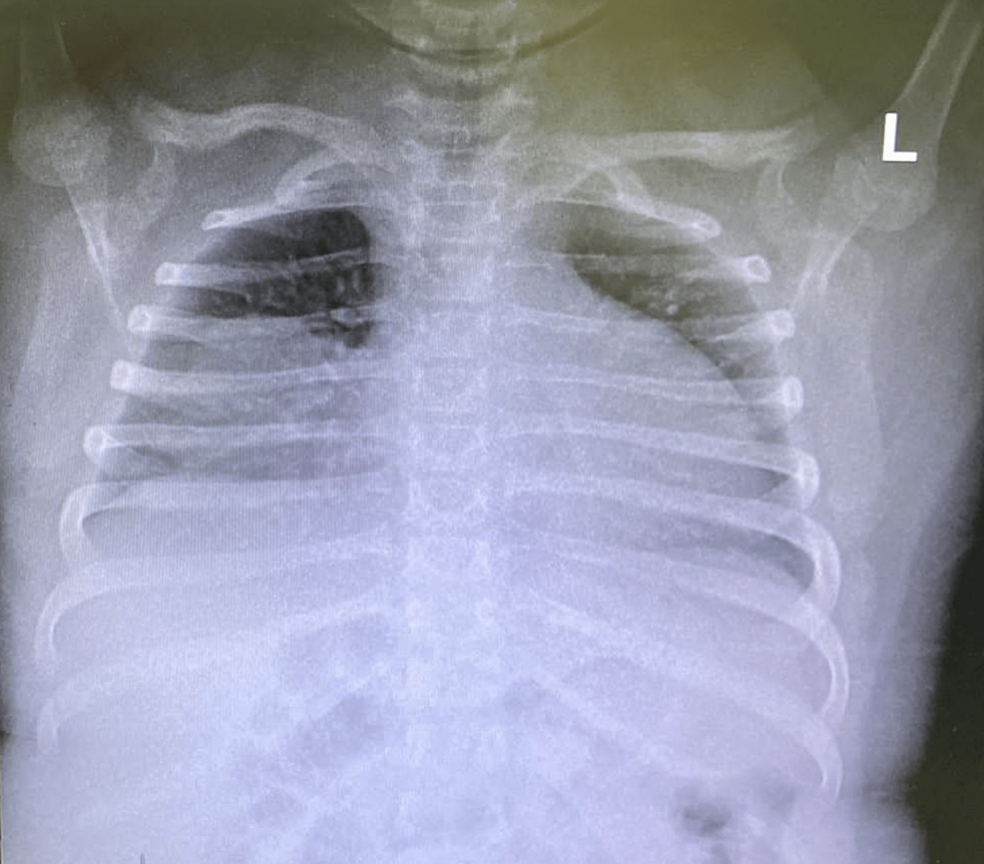
Chest X-ray showing hyperinflated lungs and a mass on the right side of the chest extending to the abdomen.

A chest X-ray showed hyperinflated lungs and a mass on the right side of the chest extending to the abdomen (Figure 1).

**Figure 2:**
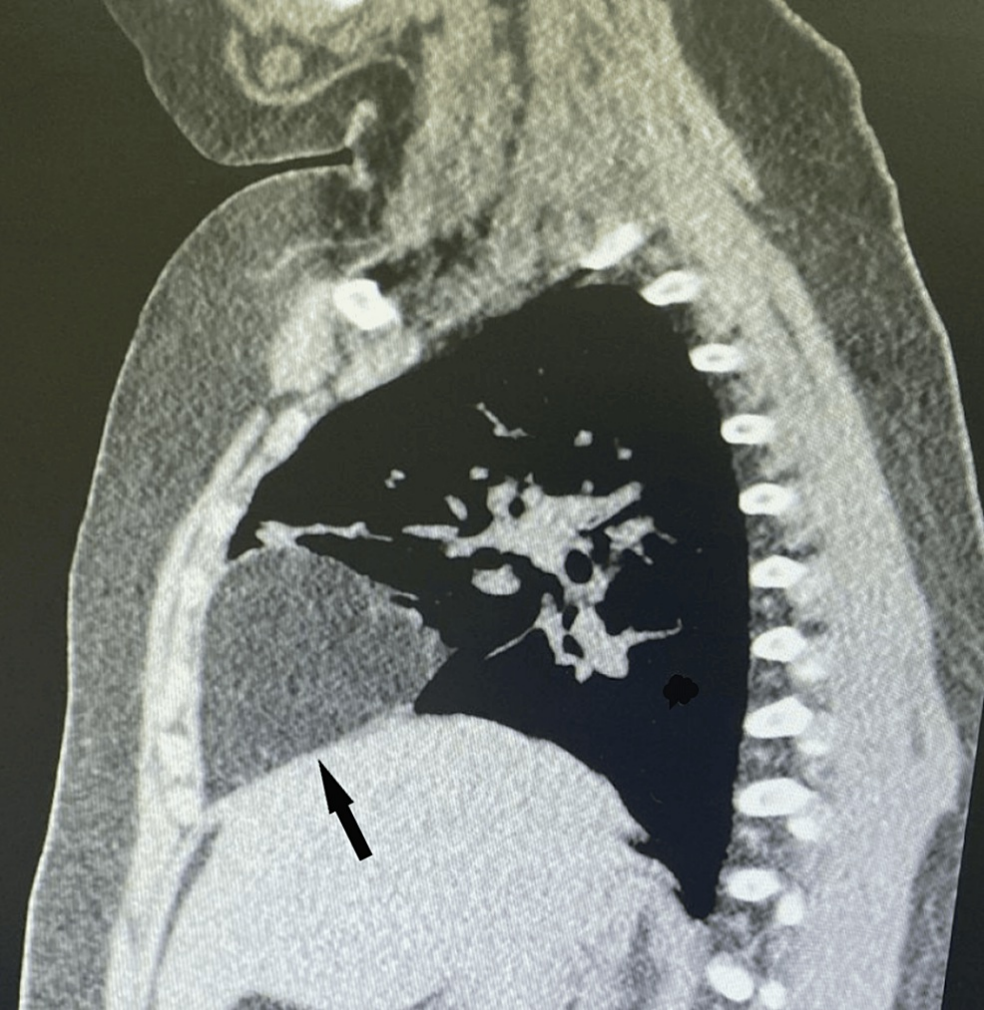
Computed tomography with contrast showing herniation of omental fat in the anterior mediastinum (arrow).

CT of the chest and abdomen with contrast was then requested to further investigate the mass. The results showed that the suspected mass was MH (Figure 2).

The Surgery Department was consulted for a hernia repair appointment, and the patient was scheduled for follow-up with ENT, cardiology, and endocrinology consultation.

## Discussion

MH is a rare form of congenital diaphragmatic hernia. Classically, it occurs on the right side [8], as seen in our patient as well. Bilateral herniation is a rare yet possible occurrence [8-10]. Children often present with a wide range of respiratory manifestations, e.g., chronic cough, dyspnea, and recurrent chest infections. Therefore, subclinical MH presentations can remain unnoticed, and its diagnosis might be delayed even to adulthood [11].

In our case, the definite diagnosis was made by CT. CT is preferred over plain chest radiographs if MH is suspected because of the radiolucency of the herniated abdominal contents. Other diagnostic modalities, including esophagogastroduodenoscopy, barium or gastrografin studies, and magnetic resonance imaging have also been reported in the literature [2]. When diagnosed, surgical repair of the defect is the gold standard treatment for MH. The fear of possible bowel incarceration and strangulation leads to the recommendation of surgery for symptomatic and asymptomatic MH cases [8].

There is an increased risk of associated congenital anomalies with MH [8,12,13]. Al-Salem [8] conducted a retrospective study on 20 infants and children with MH over a period of 18 years and reported that 70% of the studied patients had associated anomalies, with DS occurring in 15% of the patients. The reporting of MH in twins with DS by Harris et al. [14] raised the possibility that genetics may play a role in the etiology of congenital diaphragmatic hernias. In fact, Honoré et al. [12] reported a highly significant association between the occurrence of MH and DS. Pokorny et al. [1] also reported that three out of five infants with MH had DS. Witters et al. [15] recommended karyotyping MH patients, especially when other malformations are found.

Another concerning aspect regarding the occurrence of MH in DS patients is the possible association between DS and delayed diagnosis of MH. Jetley et al. [16] studied 22 patients with MH, half of them with DS. The number of patients with delayed diagnosis was notably higher in the DS group. This could be attributed to the wide spectrum of the differential diagnosis of a child with DS presenting with respiratory distress, including congenital heart disease, upper and lower respiratory tract infections, gastroesophageal reflux, and structural anomalies [17].

## Conclusions

We reported a case of an asthmatic 10-year-old DS patient who turned out to have right-sided MH. The hernia was discovered by imaging. Our case is a reminder that a child with DS can be affected by a magnitude of different disorders. MH may remain unnoticed and should be considered in the differential diagnosis of DS patients who present with respiratory distress not responding to treatment.

## References

[1] Pokorny WJ, McGill CW, Harberg FJ (1984). Morgagni hernias during infancy: presentation and associated anomalies. J Pediatr Surg.

[2] Latif Al-Arfaj A (1998). Morgagni's hernia in infants and children. Eur J Surg.

[3] Al-Salem AH (2017). Congenital Morgagni’s hernia in infants and children: a national review. Ann Pediatr Surg.

[4] Minneci PC, Deans KJ, Kim P, Mathisen DJ (2004). Foramen of Morgagni hernia: changes in diagnosis and treatment. Ann Thorac Surg.

[5] Sanford Z, Weltz AS, Brown J, Shockcor N, Wu N, Park AE (2018). Morgagni hernia repair: a review. Surg Innov.

[6] Machmouchi M, Jaber N, Naamani J (2000). Morgagni hernia in children: nine cases and a review of the literature. Ann Saudi Med.

[7] Beg MH, Rashidi ME, Jain V (2010). Morgagni hernia with Down syndrome: a rare association. Indian J Chest Dis Allied Sci.

[8] Al-Salem AH (2007). Congenital hernia of Morgagni in infants and children. J Pediatr Surg.

[9] Garofalo S, Guanà R, Schleef J (2016). Thoracic intestinal sounds in an infant with congenital bilateral Morgagni hernia. J Pediatr.

[10] Aghajanzadeh M, Khadem S, Khajeh Jahromi S, Gorabi HE, Ebrahimi H, Maafi AA (2012). Clinical presentation and operative repair of Morgagni hernia. Interact Cardiovasc Thorac Surg.

[11] Rogers FB, Rebuck JA (2006). Case report: Morgagni hernia. Hernia.

[12] Honoré LH, Torfs CP, Curry CJ (1993). Possible association between the hernia of Morgagni and trisomy 21. Am J Med Genet.

[13] Quah BS, Menon BS (1996). Down syndrome associated with a retroperitoneal teratoma and Morgagni hernia. Clin Genet.

[14] Harris GJ, Soper RT, Kimura KK (1993). Foramen of Morgagni hernia in identical twins: is this an inheritable defect?. J Pediatr Surg.

[15] Witters I, Legius E, Moerman P (2001). Associated malformations and chromosomal anomalies in 42 cases of prenatally diagnosed diaphragmatic hernia. Am J Med Genet.

[16] Jetley NK, Al-Assiri AH, Al-Helal AS, Al-Bin Ali AM (2011). Down's syndrome as a factor in the diagnosis, management, and outcome in patients of Morgagni hernia. J Pediatr Surg.

[17] Parmar RC, Tullu MS, Bavdekar SB, Borwankar SS (2001). Morgagni hernia with Down syndrome: a rare association -- case report and review of literature. J Postgrad Med.

